# Hyperprolactinemia with normal serum prolactin: “Hook effect” a concern in laboratory medicine aspect

**DOI:** 10.4103/0974-1208.74163

**Published:** 2010

**Authors:** Viroj Wiwanitkit

**Affiliations:** Wiwanitkit House, Bangkhae, Bangkok - 10160, Thailand

Sir,

I read the interesting case report by Agarwal *et al*., with a great interest.[1] There are some additional discussions on this case. It is no doubt that “all gynecologists should consider galactorrhea even in women with normal serum prolactin.”[2] The interpretation of the serum prolactin level needs careful consideration. In laboratory medicine, the false negative in prolactin level determination can be expected. A high-dose hook effect in the PRL assay that leads to falsely low serum prolactin level is mentioned in some literatures.[3–6] The hook effect should be considered in any case with a large pituitary mass.[4] A pre-dilution preparation can help in solving this specific false negative problem.[3–6]

## References

[1] Agarwal M, Das A, Singh SA (2010). Hyperprolactinemia with normal serum prolactin: Its clinical significance. J Hum Reprod Sci.

[2] Nahid E (2004). Fertility Rate with bromocriptine in infertile women with galactorrhoea and normal prolactin level. Int Congr Ser.

[3] Schöfl C, Schöfl-Siegert B, Karstens JH, Bremer M, Lenarz T, Cuarezma JS (2002). Falsely low serum prolactin in two cases of invasive macroprolactinoma. Pituitary.

[4] Al Sifri SN, Raef H (2004). The hook effect in prolactin immunoassays. Saudi Med J.

[5] Frieze TW, Mong DP, Koops MK (2002). “Hook effect” in prolactinomas: Case report and review of literature. Endocr Pract.

[6] St-Jean E, Blain F, Comtois R (1996). High prolactin levels may be missed by immunoradiometric assay in patients with macroprolactinomas. Clin Endocrinol (Oxf).

